# Characterization of physiological defects in adult SIRT6^-/-^ mice

**DOI:** 10.1371/journal.pone.0176371

**Published:** 2017-04-27

**Authors:** Victoria Peshti, Alexey Obolensky, Liat Nahum, Yariv Kanfi, Moran Rathaus, Maytal Avraham, Simon Tinman, Fredrick W. Alt, Eyal Banin, Haim Y. Cohen

**Affiliations:** 1 The Mina & Everard Goodman Faculty of Life Sciences, Bar-Ilan University, Ramat-Gan, Israel; 2 Department of Ophthalmology, Hadassah-Hebrew University Medical Center, Jerusalem, Israel; 3 Harvard University Medical School, Boston, Massachusetts, United States of America; University of Melbourne, AUSTRALIA

## Abstract

The NAD+-dependent SIRT6 deacetylase was shown to be a major regulator of lifespan and healthspan. Mice deficient for SIRT6 develop a premature aging phenotype and metabolic defects, and die before four weeks of age. Thus, the effect of SIRT6 deficiency in adult mice is unknown. Here we show that SIRT6^-/-^ mice in mixed 129/SvJ/BALB/c background reach adulthood, allowing examination of SIRT6-related metabolic and developmental phenotypes in adult mice. In this mixed background, at 200 days of age, more than 80% of the female knock-out mice were alive whereas only 10% of male knock-out mice survived. In comparison to their wild-type littermates, SIRT6 deficient mice have reduced body weight, increased glucose uptake and exhibit an age-dependent progressive impairment of retinal function accompanied by thinning of retinal layers. Together, these results demonstrate a role for SIRT6 in metabolism and age-related ocular changes in adult mice and suggest a gender specific regulation of lifespan by SIRT6.

## Introduction

The sirtuins are highly conserved NAD^+^-dependent deacetylases, homologues of the yeast Sir2 protein. They were shown to regulate lifespan in variety of organisms including mammals. In yeast, nematode and flies, an additional copy of SIR2 increased lifespan [1–3]. In mammals there are seven sirtuins, SIRT1 to SIRT7; among them SIRT1 is the most investigated. Mice over-expressing SIRT1 showed improved healthspan, including lower levels of DNA damage, better glucose tolerance and fewer spontaneous carcinomas and sarcomas. Whole body overexpression of SIRT1 did not extend lifespan [4], but brain specific overexpression in mice did increase lifespan [5].

In the last few years SIRT6 was established as the main mammalian sirtuin which regulates lifespan. SIRT6 is predominantly a nuclear sirtuin which, besides deacetylation [6, 7], also catalyzes ADP-ribosylation [8] and deacylation of long chain fatty acyls [9]. SIRT6-deficient 129/SvJ mice are small, express severe metabolic defects, and by 2–3 weeks of age develop abnormalities usually associated with aging. These include profound lymphopenia, loss of subcutaneous fat, lordokyphosis, and eventually death at about 4 weeks [10]. Serum IGF-1 and glucose levels are both severely reduced in SIRT6^-/-^ mice at the age of 24 days [10]. In addition, SIRT6 deficiency caused major retinal transmission defects concomitant to changes in expression of glycolytic genes and elevated levels of apoptosis in inner retina cells [11]. An additional study that examined the effect of SIRT6 deficiency in a mixed genetic background showed a similar set of systemic phenotypes including the fact that the majority of these mice do not exceed five weeks of age [12]. SIRT6 is required for glucose homeostasis and maintenance of normal IGF-1 levels. Indeed, SIRT6 was found to regulate the expression of glycolytic genes, including the glucose transporter GLUT1 [13]. In the absence of SIRT6, GLUT1 levels increase, resulting in increased glucose uptake and glycolysis activation. SIRT6 also functions at chromatin to attenuate NF-κB signaling via interaction with the NF-κB RELA subunit, and deacetylates H3K9 at NF-κB target gene promoters [7].

SIRT6 is an important key player in different DNA repair pathways and it is crucial for genomic stability [8, 10, 14–16]. *In vitro*, SIRT6 depletion leads to telomere dysfunction with end-to-end chromosomal fusions and premature cellular senescence [14, 17]. Moreover, SIRT6 is required for the stable association of telomeric chromatin with WRN, a protein that is mutated in the premature aging disease Werner syndrome [18]. These findings suggest that SIRT6 is important in telomere maintenance. Recently SIRT6 was shown to be regulated by Lamin A, another protein whose mutant form, Progerin, causes premature aging disease in humans [19]. Taken together, this data suggests that SIRT6 dysfunction may play an important role in human premature aging.

SIRT6 ability to control lifespan and healthspan is linked to a number of age-related diseases. We found transgenic mice over-expressing exogenous SIRT6 (MOSES) to be protected against the physiological damages of diet-induced obesity [20] including accumulation of triglycerides and LDL cholesterol in the serum. In addition, SIRT6 regulates healthy liver via the repression of SREBP1/2, two transcription factors that control triglycerides and cholesterol synthesis and via repressing miR122, the most abundant microRNA in the liver [21], [22]. Indeed, MOSES males have a significantly longer lifespan than their wild-type (WT) littermates. Moreover, MOSES mice have reduced IGF-1 levels in sera and IGF-1 signaling was significantly reduced in white adipose tissues (WAT) [23]. In addition, SIRT6 has a pivotal role in carcinogenesis [24]. It was found to be a tumor suppressor by controlling Myc transcriptional activity [24, 25].

SIRT1 is another sirtuin whose deficiency also causes a severe phenotype. Interestingly, the effect of SIRT1 deficiency is strain dependent, especially as relates to mice survival. Whereas SIRT1-deficient mice on 129/SvJ-CP background were smaller and invariably died within 1 month after birth, mice on 129/CD1 mixed background more often survived to adulthood. In 129/SvEv/C57BL/6 background, about 90% of mutant mice died at perinatal or early postnatal stages, and the remaining died about 3 months after birth [26, 27]. In another study only 1% of the 129/SvEv/FVB background and 9.3% on the 129/SvEv/FVB/Black Swiss background survived to adulthood [28]. These observations suggest the reported phenotypes of 129/SvJ SIRT6^-/-^ mice might be strain dependent as well. Due to the very short lifespan of the various SIRT6^-/-^ animal models which usually do not exceed five weeks of age, our knowledge regarding the role of SIRT6 in adult and aging mice is still limited. Indeed, all of the reported phenotypes for SIRT6 deficiency are based on experiments that were done in mice younger than two months old. Moreover, given that the characterization of SIRT6 effects was done in young mice it is not known if any of the reported phenotypes are gender specific. Therefore, two critical pieces are missing in this puzzle: what would be the effect of SIRT6 deficiency in adult mice and whether this effect is gender specific. Here we show that SIRT6^-/-^ mice in a mixed 129/SvJ/BALB/c background display increased survival that allows us to explore new SIRT6-related metabolic and developmental phenotypes in adult, aging mice.

## Materials and methods

### Mice and MEFs

The ethic committee of Bar Ilan University (Animal Care and Use Committee (IACUC) approved this study. Mice were kept under specific pathogen free and 12 h light ⁄ dark conditions. SIRT6+/- 129/SvJ mice were mated with BALB/c mice (Jackson). Breeding of SIRT6 heterozygote F1 generation resulted in SIRT6^+/+^, SIRT6^+/-^ and SIRT6^-/-^ 129/SvJ/BALB/c mice.

The previously published findings that 129/SvJ SIRT6 deficient mice die up to four weeks prematurely was taken into consideration and addressed in the approved protocol by the above-mentioned committee. Thus, the mixed background mice who survived significantly longer were monitored daily. The specific criteria to performing euthanasia were disease, severe injury and apathy. There were no unexpected deaths due to apparent diseases or injuries. At the time of the examination, animals that died without euthanasia did not exhibit any signs of distress or signs of being moribund prior to their death. In addition, no apparent cause of death was found in these mice.

General microbial examinations were performed routinely by the animal research facility staff. For GTT and ITT, Esracain cream (Lidocaine 5%, Rafa, Israel) was used as topical analgesic. Before ERG procedures mice were anesthetized by intraperitoneal injections of a mixture of ketamine (Bedford Laboratories, Bedford, OH) and xylazine (VMD, Arendonk, Belgium), with doses suitable to their body weight. After completion of ERG recordings, while still anesthetized, mice were sacrificed by cervical dislocation. Mice that were not examined by ERG recordings and did not die naturally were put to death by CO_2_.

Mouse Embryo Fibroblasts (MEFs) were isolated 13 days after observation of vaginal plugs. Cells were plated into DMEM media supplemented with 10% fetal bovine serum (FBS).

### Metabolic measurements

Body composition was analyzed by Dual-Energy X-ray Absorptiometry (DEXA) using a Lunar PIXImus densitometer. For glucose tolerance testing (GTT), mice were subjected to overnight fasting and injected intraperitoneally (IP) with 2g of glucose per kg of body weight. Blood glucose was measured prior to glucose administration and 15, 30, 60, 90, and 120 min after using an Ascensia Elite glucose meter (Bayer). For the insulin tolerance test (ITT), mice were fasted for 6h, injected IP with 0.75 U insulin (Eli Lilly) per kg of body weight, and blood glucose levels were measured before and 15, 30, 60, 90 and 120 min after injection. HOMA of insulin resistance (HOMA-IR) was calculated using the equation: fasting serum insulin (μU/ml) × fasting plasma glucose (mmol/l)/22.5. Serum insulin was measured by ELISA (Crystal Chem).

### Quantitative real time PCR

Total RNA was extracted using RNeasy kit according to the manufacturer’s protocol. cDNA was generated using SuperScript Vilo (Invitrogen). Quantitative real-time PCR was performed in triplicate using Absolute blue SYBR Green (Thermo) in a Chromo4 instrument cycler (Bio-Rad). Ct values were normalized to 18S.

Primer sequences were as follows: Glut1, 5'-TCAACGAGCATCTTCGAGAAGGCA-3' and 5'-TCGTCCAGCTCGCTCTACAACAAA-3'; 18S, 5'-AGGAATTGACGGAAGGGCAC-3' and 5'-GTGCAGCCCCGGACATCTAAG-3'.

### Histology

Tissues were fixed in 4% buffered formaldehyde, embedded in paraffin, sectioned and stained with hematoxylin and eosin. The histology analysis of skin sections was done by Patholab, Israel.

### Electroretinography (ERG)

Full field ERG was performed in anesthetized animals following overnight dark adaptation using a Ganzfeld dome and a computerized system (Espion E^2^, Diagnosys LLC, Littleton, MA). Pupils were dilated with 1% tropicamide and 2.5% phenylephrine and local anesthetic drops (benoxinate HCl, 0.4%; all ocular drops from Fisher Pharmaceuticals, Tel-Aviv, Israel), were administered prior to placing gold-wire active electrodes on the central cornea. A reference electrode was placed on the tongue and a needle ground electrode was placed intramuscularly in the hip area. Dark-adapted rod and mixed cone-rod as well as light-adapted 16Hz cone flicker responses to a series of white flashes of increasing intensities (0.00008–9.6 cd•s/m^2^) were recorded. All ERG responses were filtered at 0.3–500Hz, and signal averaging was applied.

### Ocular histology and measurements of outer nuclear layer (ONL) thickness

Eyes were enucleated, fixed in Davidson solution, embedded in paraplast, and serially cut in 4 μm sections through the center of the optic nerve. For descriptive histology and quantitative analysis, sections were stained with hematoxylin and eosin. All observations and photography were performed using an Olympus BX41 microscope equipped with a DP70 digital camera. Image processing and quantification were performed using Adobe Photoshop CS3 and ImagePro Plus 6.0 software.

Thickness of the ONL was measured as previously described [29]. Briefly, ONL thickness was measured three times in the central part of each hemiretina and results from the corresponding retinal areas were averaged.

### Statistical analysis

Results are expressed as mean ± SEM. P-values were calculated by unpaired Student’s t-tests or one-way ANOVA using SPSS Statistics 17.0 software.

## Results

SIRT6^+/-^ 129/SvJ mice [10] were mated with BALB/c mice to examine the effect of SIRT6 deficiency in this hybrid background. SIRT6 deficiency was validated in MEFs, liver and muscle (S1 Fig). Breeding of the resulting SIRT6 heterozygote F1 generation obtained SIRT6^+/+^, SIRT6^+/-^ and SIRT6^-/-^ 129/SvJ/BALB/c mice. In 182 newborns, genotype segregation did not show the expected Mendelian ratio of 1:2:1 (Table 1), with a higher prevalence of WT mice (χ^2^ test analysis, p< 0.05). Interestingly, the genotype ratio in embryos at day 13.5 (E13.5) was as expected from mendelian genetics, suggesting that the change in the ratio observed in born mice might be caused by selective and increased prenatal mortality of SIRT6^+/-^ and SIRT6^-/-^ embryos between E13.5 and birth (Table 1). Therefore, in the 129/SvJ/BALB/c background, partial or complete absence of SIRT6 had a significant effect on prenatal survival.

**Table 1:** Genotypes of progeny from crosses of F1 SIRT6+/− mice.

Stage	Percent (and number) of offspring		
	WT	HET	KO
**E13.5**	30.4% (14)	50% (23)	19.6% (9)
**Born**	34% (62)	43.4% (79)	22.5% (41^1^)

^1^Within 15 females and 26 males, only one male died before 30 days of age.

**Fig 1:**
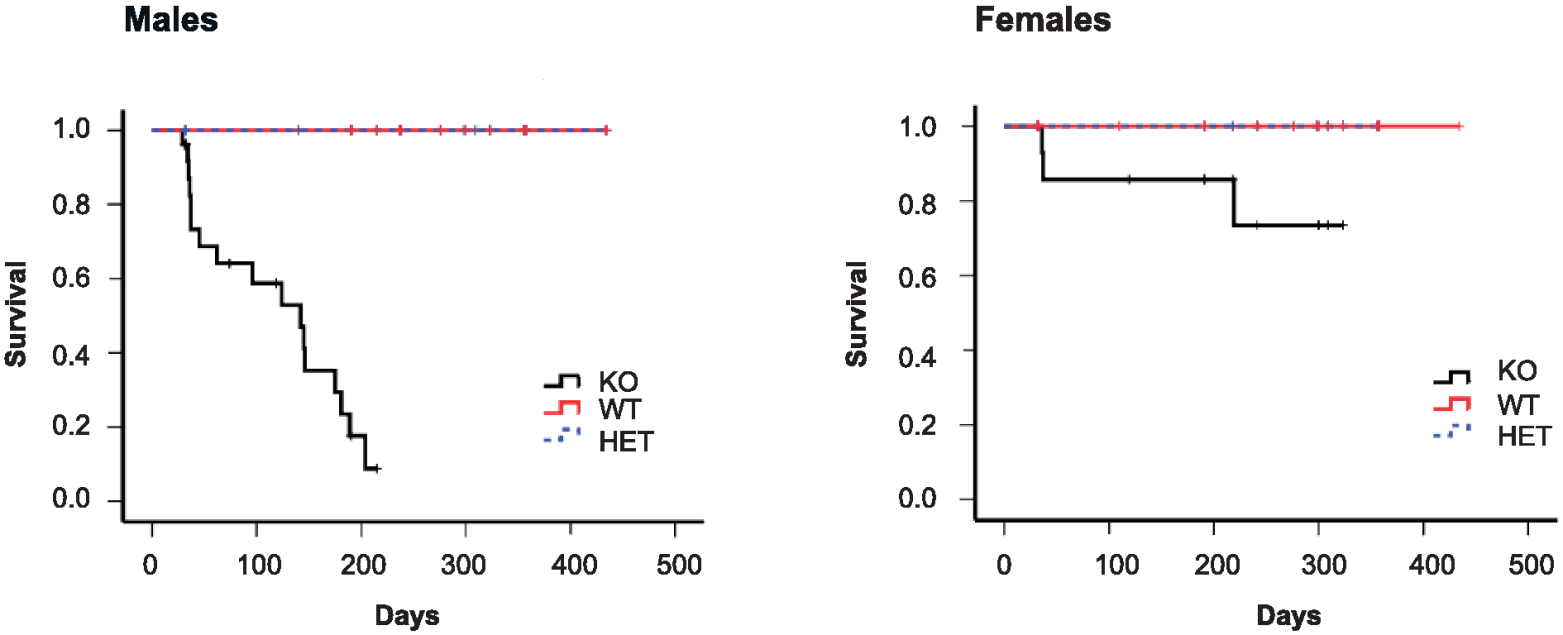
Gender specific effect of SIRT6 deficiency on mouse survival. Kaplan—Meier survival curves of WT, HET and KO male (left panel) and female (right panel) mice in 129/SvJ/BALB/c background. 90% of KO male mice died before 200 days of age and more than 75% of KO female mice survived over 300 days of age. Males, n = 23–39 per group; Females, n = 14–40 per group.

The survival of male and female 129/SvJ/BALB/c mice was compared between SIRT6 KO and WT littermates. In both genders, death of KO mice began to occur within 30 days. Yet, whereas the median survival time of SIRT6 KO male mice was 124 days and 90% of them died before 200 days of age, more than 75% of the female KO mice survived over 300 days of age (Fig 1). These findings show a gender specific effect of SIRT6 on mouse survival.

**Fig 2:**
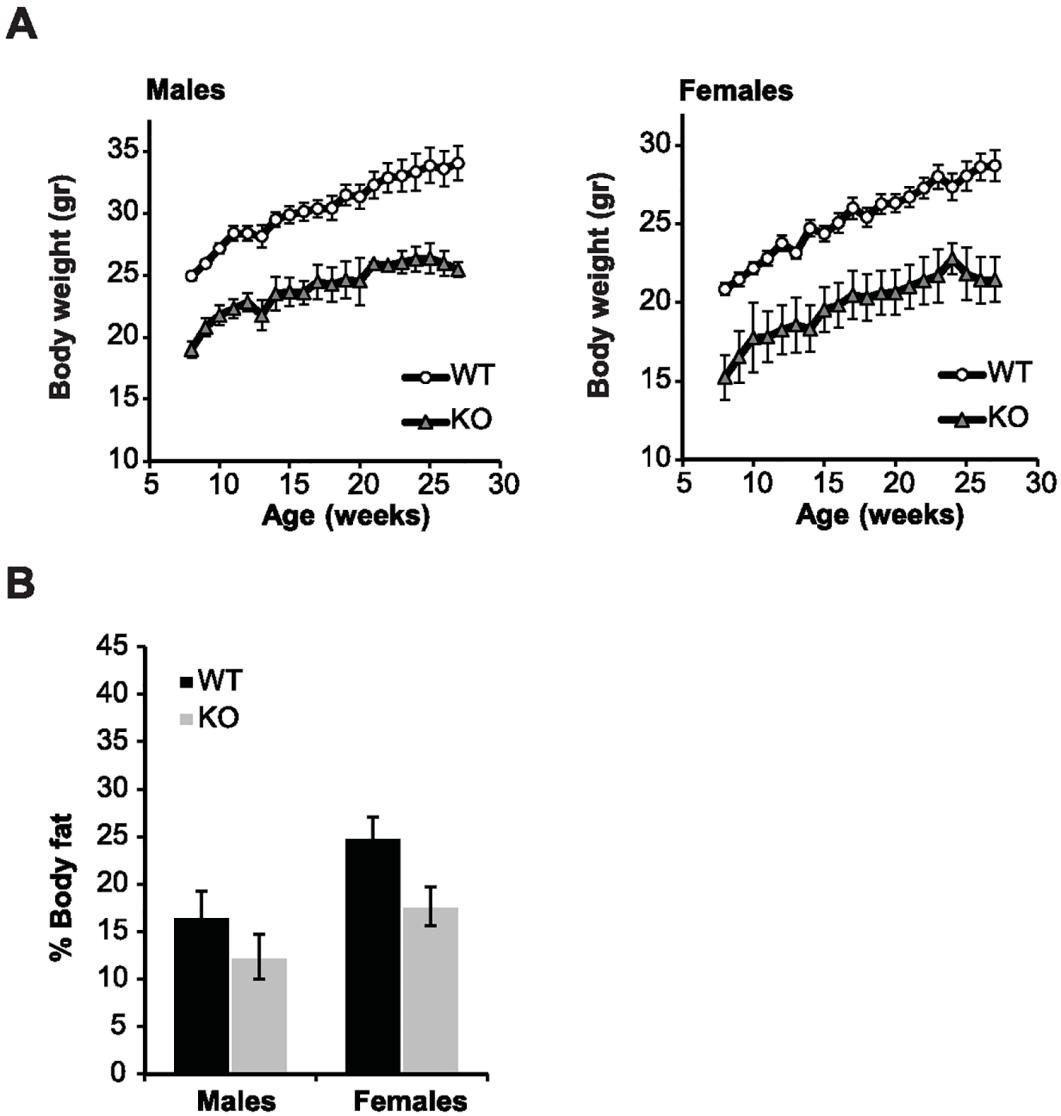
Effect of SIRT6 deficiency on body weight and adiposity. (A) Average body weight of WT and KO male (left panel) and female (right panel) mice. In both genders, the weight of SIRT6 KO mice was significantly lower as compared with age-matched WT animals. (B) KO mice showed a trend towards lower percentage of total body fat in comparison to their WT littermates. The percentage of total body fat was measured at 6–8 months of age. Results are presented as mean ± SEM. n = 3–14 per group.

Next, the effect of SIRT6 deficiency on metabolism in this hybrid background was examined. In comparison to their WT littermates, the body weight of SIRT6 KO mice was significantly lower in both genders (Fig 2A), as has been seen in a previous study [10]. Differently, both male and female KO mice did not show significant change in total body fat in comparison to their WT littermates (Fig 2B), only a trend. Such a change was seen in another mixed genetic background [12]. Skin sections stained with hematoxylin and eosin (H&E) revealed loss of subcutaneous fat in KO male mice (S2 Fig), similar to changes in subcutaneous fat that have been previously reported [10].

**Fig 3:**
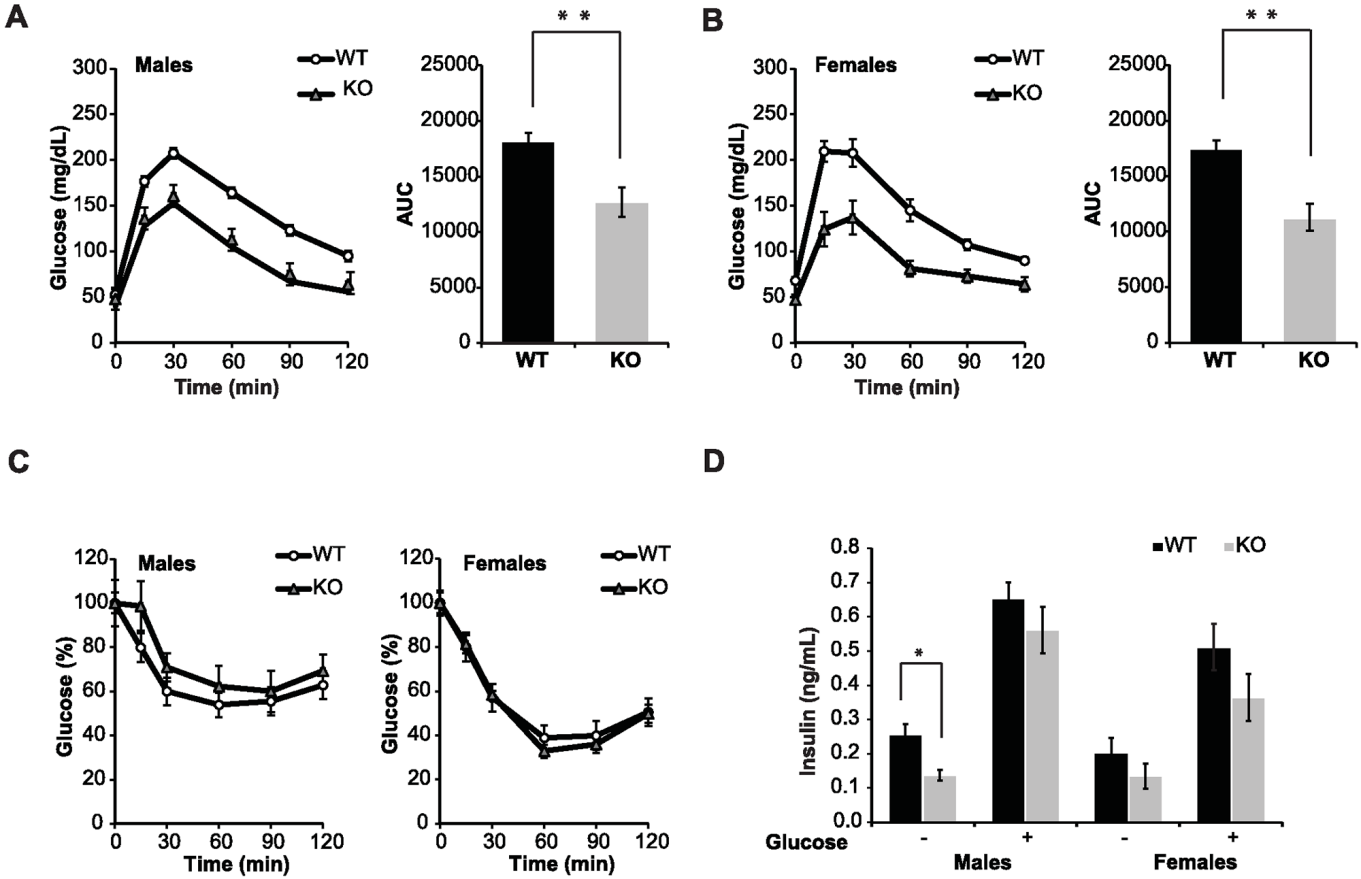
Increased glucose uptake in SIRT6 deficient mice. Glucose tolerance test (GTT) in WT and KO male (A) and female (B) mice. Area under curve (AUC) is shown on the right for each GTT. Both male and female KO mice show increased glucose uptake in comparison to WT mice. (C) Insulin tolerance test in male (left panel) and female (right panel) mice showed no significant difference between genotypes. (D) Insulin blood levels after glucose stimulation as determined by ELISA. All assays were done at 3–4 months old. Results are presented as mean ± SEM. n = 5–14 per group. * *P* < 0.05, ** *P* < 0.01

The effect of SIRT6 deficiency on glucose homeostasis was examined initially through a glucose tolerance test (GTT) in mice fed with chow diet. Significantly, increased glucose uptake was found in both male and female KO mice during the 2 hours after glucose injection as compared to WT littermates (Fig 3A and 3B). The calculated homeostasis model assessment (HOMA) measure of insulin resistance was lower in KO mice as compared with WT animals (0.41±0.07 vs. 0.85±0.13, *P* = 0.033 for males and 0.32±0.22 vs. 0.82±0.12, *P* = 0.07 in females). The observed increased glucose uptake in KO mice could result from increased insulin sensitivity, an increase in insulin secretion or from insulin independent increased glucose uptake. In order to examine whether there is a change in insulin sensitivity, an insulin tolerance test (ITT) was performed. No significant difference was found between the experimental groups (Fig 3C). In order to explore the possibility that the KO mice secrete more insulin in response to glucose stimulus, we measured insulin blood levels after glucose stimulation (GSIS). As seen in Fig 3D, the KO mice secreted similar levels of insulin upon glucose stimulation in comparison to their WT littermates. Basal insulin level is known to be significantly low in SIRT6 deficient mice [12, 13]. As seen in Fig 3D, basal insulin levels are significantly lower only in KO male mice whereas in females there was no significant change, although both male and female KO mice presented higher glucose uptake compared to their WT littermates. Altogether these findings suggest that in this mixed background the increased glucose uptake in SIRT6 KO mice is insulin independent.

**Fig 4:**
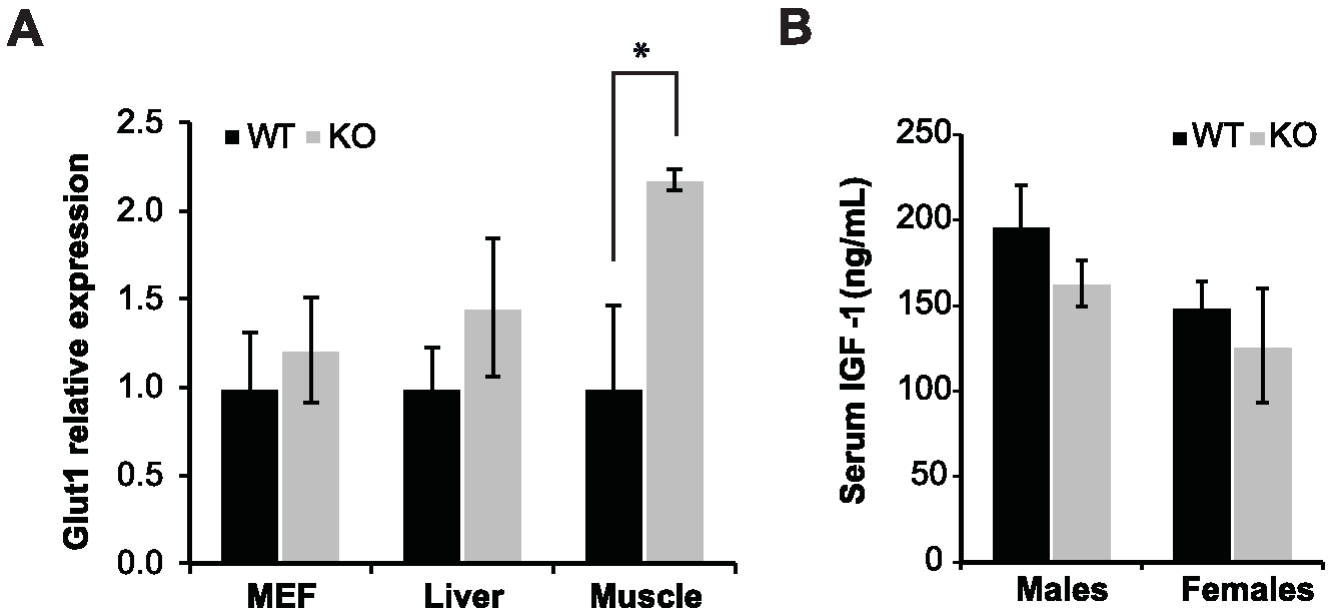
Effect of SIRT6 deficiency on GLUT1 expression and serum IGF-1 levels. **(A)** Relative expression of GLUT1 in MEF's, liver and muscle of WT and KO male mice as determined by quantitative real-time PCR. **(B)** Serum IGF-1 levels of WT and KO male and female mice. All measurements were done at 6–8 months of age. Results are presented as mean ± SEM. n = 5–14 per group. * *P* < 0.05.

GLUT1 is an insulin independent glucose transporter. Thus, the levels of GLUT1 in these mice were measured. First, GLUT1 RNA levels were measured in mouse embryonic fibroblasts (MEF's), liver and muscle of WT and SIRT6 KO mice. SIRT6 protein deficiency in these KO tissues and cells, was validated by western blot analyses (S1 Fig). In comparison to their WT littermates, no significant difference in the RNA expression levels of GLUT1 was found in liver and MEFs of SIRT6 KO 129/SvJ/BALB/c mice (Fig 4A). In addition, no difference in the levels of GLUT1 in the membrane of SIRT6 KO MEFs in this background was found (S3 Fig). However, in muscle, expression levels of GLUT1 in SIRT6 KO 129/SvJ/BALA/c male mice (Fig 4A) was twofold higher than in WT mice (*P* < 0.05). As the muscle is a major organ of glucose consumption, this could explain the higher glucose uptake in these mice. Interestingly, these findings are in concordance with a previous report showing similar GLUT1-dependent increased glucose uptake in SIRT6 ^-/-^ 129/SvJ mice [13] and unlike SIRT6 ^-/-^ 129/Black Swiss/FVB mice in which the higher glucose uptake is controlled by insulin [12].

In SIRT6 deficient 129/SvJ mice, IGF-1 levels were decreased to a level similar to mice with liver specific IGF-1 deficiency [10]. Thus, we measured IGF-1 levels in the sera of SIRT6 KO and SIRT6^+/+^ 129/SvJ/BALB/c mice. In both male and female KO mice, no significant decrease in IGF-1 levels was found (Fig 4B).

**Fig 5:**
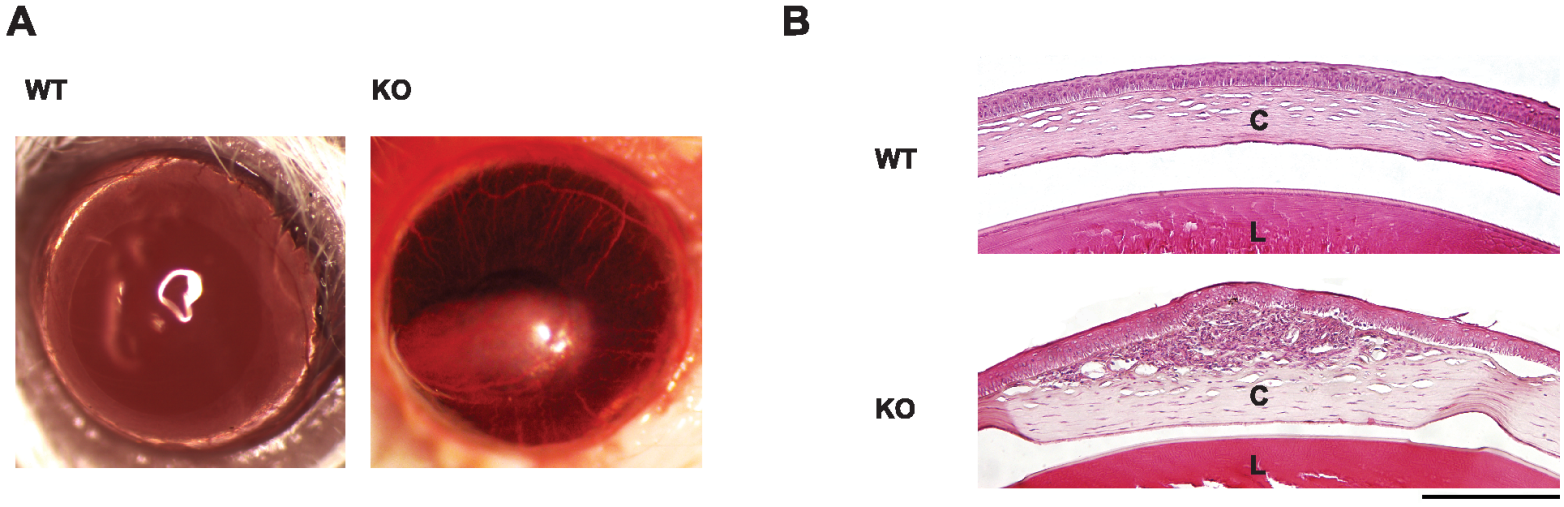
Corneal changes in SIRT6 deficient mice over time. (A-B) Photographs and histological sections of corneas in 10-month-old WT and KO mice. Corneas of normal WT mice are fully transparent, with no ingrowth of blood vessels (A, left panel). In contrast, from 6 months of age, corneal scarring and vascularization were often present in KO mice eyes (A, right panel). Histological section in KO mice (B, lower panel) shows corneal thickening, stromal edema, inflammation and scarring that are not present in WT mice of the same age (B, upper panel). B: Paraffin embedded sections stained with hematoxylin and eosin; C- Cornea; L-Lens; original magnification x10; Scale bar = 200μm.

**Fig 6:**
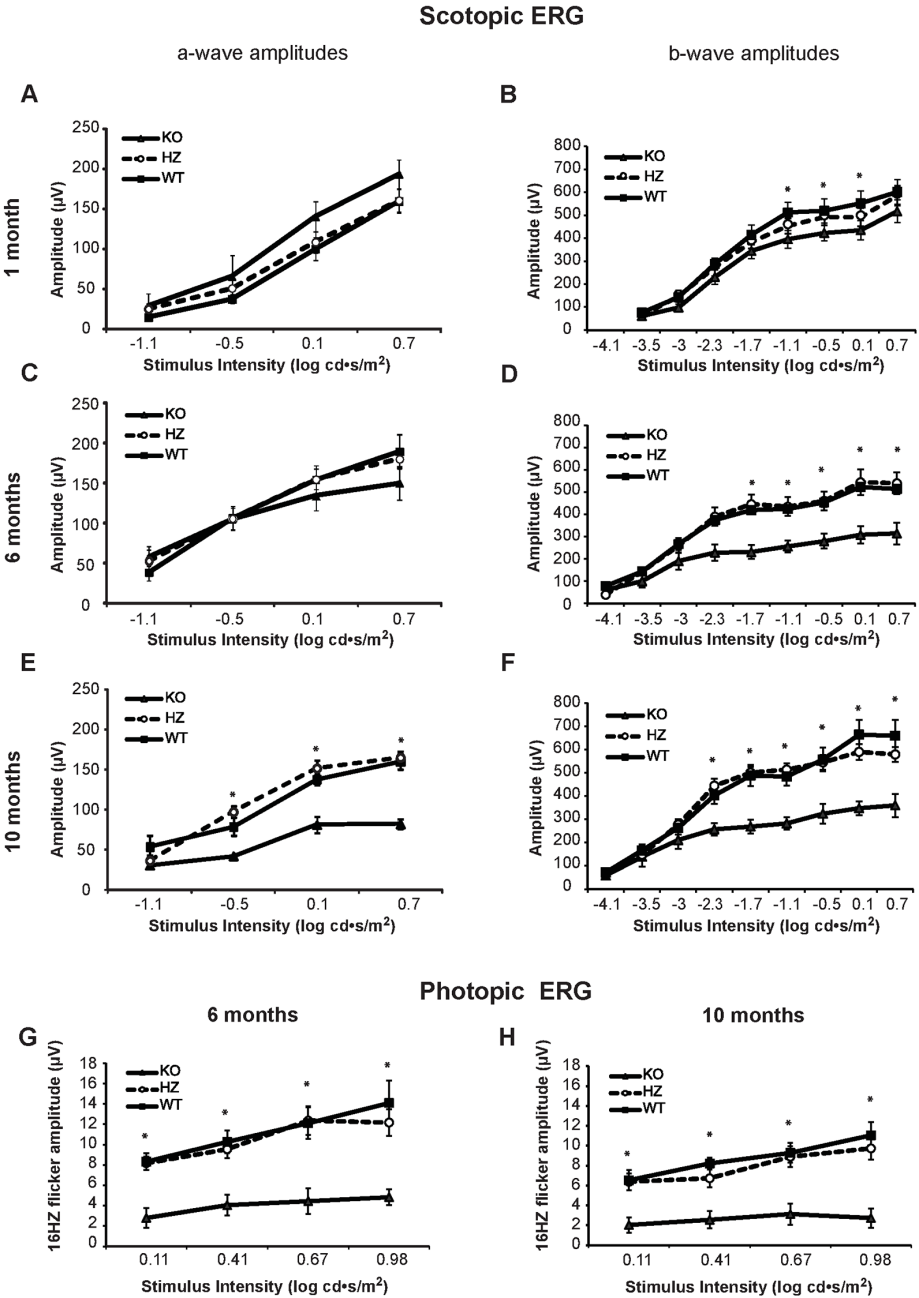
Retinal function in SIRT6 deficient mice. Scotopic (A-F) and photopic (G, H) full-field electroretinography (ERG) recordings may suggest accelerated aging in KO eyes. At one month of age, dark-adapted ERG responses to a series of increasing white flash intensities showed preserved a-wave amplitudes in KO as compared to WT and HET eyes (A), while a trend towards lower amplitudes was observed in the scotopic b-wave amplitudes, attaining statistical significance at 3 high intensities (B). At the age of 6 months, scotopic a-wave responses at higher intensities showed a trend towards lower amplitudes (C), and b-wave amplitudes were markedly reduced (D). At 10 months, both a- and b-wave amplitudes in KO mice were markedly reduced as compared to WT and HET mice (E, F). Light adapted 16Hz flicker responses were also markedly reduced in KO mice at 6 and 10 months (G, H). ERG responses were similar in WT and HET mice at all ages. Results are presented as mean ± SEM; n = 6–9 for WT, n = 7–8 for HET, and n = 4–5 for KO mice. * *P* < 0.05.

**Fig 7:**
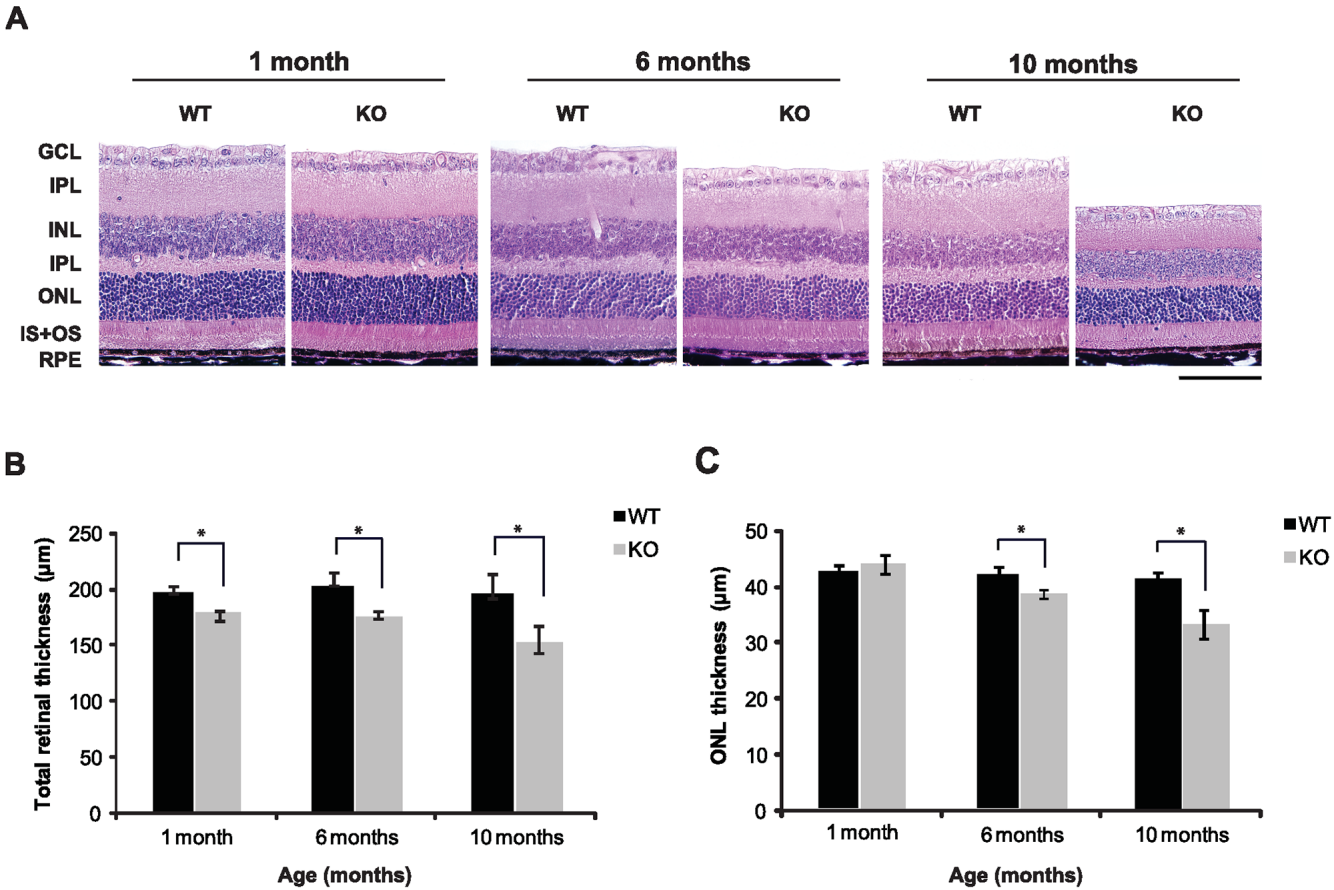
Retinal structural changes well correlated with the ERG findings. (A) Representative retinal sections stained with hematoxylin and eosin demonstrate marked decrease of retinal thickness in KO eyes by the age of 6 months with further thinning by 10 months. Original magnification x20; Scale bar = 100μm. (B) Throughout the experiment, total retinal thickness was reduced in KO eyes as compared with WT littermates. (C) Thickness of the outer nuclear layer (ONL, containing the photoreceptor nuclei) did not differ between WT and KO mice at the age of 1 month, but by the age of 6 and 10 months, the ONL was significantly thinner in animals lacking SIRT6 activity. Results are presented as mean ± SEM; n = 5–7 for WT, n = 4–5 for KO mice. * *P* < 0.05.

Corneal injury which we observed in adult KO mice prompted us to study the eye in this model more closely. At one month of age, SIRT6 KO mice had normal corneal appearance (not shown). However, by 5–6 months, severe corneal injury was often observed in SIRT6 KO mice including corneal scarring, vascularization, signs of stromal edema and inflammation (Fig 5A right panel and Fig 5B lower histological section). Assessment of retinal function by full field electroretinography (ERG) showed differences in amplitudes of dark- and light-adapted responses between WT and KO mice (Fig 6). At one month of age, SIRT6 KO mice showed preserved a-wave amplitudes in KO as compared to WT and HET eyes (Fig 6A), while a trend towards lower dark-adapted b-wave ERG amplitudes was observed, attaining statistical significance at high stimulus intensities (Fig 6B). At this time point no differences in outer nuclear layer (ONL, photoreceptor nuclei layer) thickness were found between WT and SIRT6 KO as assessed by quantitative histological techniques (Fig 7A and 7C). However, by six months of age, scotopic a-wave responses at higher intensities showed a trend towards lower amplitudes (Fig 6C) and b-wave amplitudes were significantly reduced in SIRT6 KO animals as compared with WT and HET mice (Fig 6D). Impairment of scotopic retinal function persisted through 10 months of age (Fig 6E and 6F). Light-adapted cone responses were also markedly affected at six and ten months of age, being 2.5-3-fold lower in SIRT6 KO mice (Fig 6G and 6H). This was accompanied by thinning of the retina and its layers (Fig 7A and 7B, S4A and S4B Fig). Measurements of ONL thickness correlated with the functional ERG findings: at 6 months of age, ONL thickness was significantly reduced in the central retina of SIRT6 KO as compared with WT mice with further thinning by 10 months (Fig 7C).

## Discussion

SIRT6 deficient 129/SvJ mice display a broad-range of phenotypes and die within 4 weeks after birth [10]. Therefore, our knowledge regarding the effect of SIRT6 in adult mice is lacking. To address this, and to examine whether SIRT6 phenotypes are strain and/or age specific, we generated SIRT6 KO 129/SvJ/BALB/c mice. These KO mice on a mixed background survive to adulthood, thus allowing the opportunity to explore new SIRT6 related metabolic and developmental phenotypes unique to adult mice. In comparison to their WT littermates, SIRT6 KO mice had a gender-dependent decreased lifespan, significantly lower body weight, and faster glucose uptake. In addition, ocular changes were noted, including degradation of retinal function and structure that may reflect accelerated aging.

Different mouse models of SIRT1 deficiency showed a strain-dependent set of phenotypes [26, 27]. Similarly, deletion of Sir2 in yeast also results in different phenotypes in different backgrounds [3, 30]. In contrast to previously published SIRT6 ^-/-^ mouse models, our mixed background animals have a biased mendelian ratio between WT, SIRT6^+/-^ and SIRT6^-/-^. The SIRT6 deficient mice are not hypoglycemic, do not show lower IGF-1 levels, survive to adulthood and have gender specific effects on lifespan. Moreover, in contrast to 129/Black Swiss/FVB background animals, our mice have normal insulin-dependent glucose uptake. This strain-dependent variability may stem from the central role of deacetylases that regulate the transcription of many genes, and therefore the observed phenotypes should be considered to be a combinatorial outcome. SIRT6 as a global histone H3 K9 and H3K56 deacetylase nicely fits into such a model.

Interestingly, the newborn genotype segregation was not a Mendelian ratio of 1:2:1 (Table 1). Assuming that no death is expected in WT newborn, the number of HET newborn (73) was only 59% of the expected (124 HET newborn). Likewise, based on the WT newborns, the number of SIRT6 KO newborn is also only 66% of expected. This change in genotypes segregation was significant (*P*<0.05) whereas similar analysis on the genotype ratio of E13.5 embryos did not show a significant change in the ratio (Table 1). Therefore, SIRT6 deficiency or haplodeficiency results in significantly higher prenatal death rates starting after day E13.5.

A major advantage of the current mouse model over previously published models is that it allows examining the role of SIRT6 deficiency in adult mice. 129/SvJ mice deficient for SIRT6 die prematurely and it is therefore impossible to follow the role of SIRT6 in mice older than 4 weeks in this background. Another published model of SIRT6 deficiency in mixed background results in higher survival rates of KO mice, but in this study, the median lifespan was also about 4 weeks of age with a set of physiological defects similar to those observed in the 129/SvJ background [10, 12]. Due to the young age of the examined animals in both of these SIRT6 KO models, it is not clear if SIRT6-related pathologies are developmental or indeed representative of age-related degenerative diseases. Therefore, our knowledge regarding the role of SIRT6 in adult mice is very limited. Here, we were able to identify SIRT6 effects in mature mice up to the age of 10 months: the metabolic effects, including faster glucose uptake; the male-specific shortening of lifespan and an age-dependent impairment of retinal function accompanied by retinal thinning. Importantly, Xiao and his colleagues also reported some phenotypes for adult SIRT6 mice. However, in that report, only few mice survived for longer than five weeks and therefore these mice in fact represent the exceptions of their experimental conditions.

With regard to the metabolic effects of SIRT6 deficiency, the higher glucose uptake which appears in SIRT6 KO mice is a constitutive phenotype that was present in the two mouse models examined so far [12, 13]. Similar to these studies, 129/SvJ/BALB/c males and females examined in the present study have higher glucose uptake in KO mice [13]. In our SIRT6 KO model, as also reported in the 129/SvJ SIRT6 KO mice, an increase in peripheral levels of GLUT1 as measured in muscle seems to be the main cause of this phenotype. ITT and GSIS results suggest that this higher glucose uptake is insulin independent, differing from the study of Xiao et al. that examined SIRT6 deficiency in another mixed background in which insulin dependent hypoglycemia was reported [12]. Interestingly, in contrast to a previous study, no difference was found in the levels of GLUT1 in SIRT6 KO vs SIRT6 WT MEF's isolated from 129/SvJ/BALB/c mice.

The male-specific effect of SIRT6 deficiency on lifespan observed in the present study may correlate with the observation that SIRT6 overexpression in mixed background increased lifespan exclusively in male mice. Lack of SIRT6 similarly affected the body weight, insulin levels after glucose stimulus and GTT in males and females. Therefore the reason that SIRT6 deficient male mice have a shorter lifespan than females in 129/SvJ/BALB/c background remains unclear for now. In addition, 129/SvJ/BALB/c KO male mice showed a decrease in subcutaneous fat similarly to 129SvJ background KO mice. Reduced subcutaneous fat is a well-known marker of aging and was found in various mouse models that manifest premature aging.

Apart from the metabolic abnormalities identified in the SIRT6 KO mice, interesting changes also occurred in the eyes of affected mice including corneal injury and degradation of retinal function and structure over time. In many ways, the retinal changes resemble those seen during aging in normal mice, but appear at much earlier ages. Silberman et al [11] recently reported that young SIRT6 KO mice in 129/SvJ background have attenuated ERG amplitudes, but retinal structure was preserved at this age. In this study animals only up to 1 month of age were examined at a single time point, suggesting that the retinal dysfunction observed is not necessarily degenerative. As can be seen in Fig 6, in WT and HET animals ERG amplitudes decrease between the ages of 6 and 10 months but the reduction is much more pronounced in the 129/SvJ/BALB/c Sirt6 KO mice. At the age of 6 and 10 months, dark-adapted ERG responses to higher stimulus intensities were 40–50% lower in SIRT6 KO mice than in WT. This level of attenuation, when due to aging, was reported to occur in normal B6D2F1/J mice only at the age of 2.5 years as compared to 4 months [31]. In another study, Li et al. found that in C57BL/6 mice amplitudes of the dark-adapted ERG b-wave decreased by about 15% at the age of 6 months and by about 32% by the age of 12 months as compared with 2 month old animals [32]. The decrease noted in SIRT6 KO by 6 and 10 months exceeds these levels, but interestingly, both the functional and the structural degradation are much less severe than those seen in genetically-determined retinal degenerations such as in rd10 mice [33].

Our results may suggest a role for SIRT6 in maintenance and function of retinal cells over time. SIRT6 was shown to regulate genome stability and resistance to oxidative injury caused by H_2_O_2_ [10]. Therefore, one can predict that in the absence of SIRT6 there will be increased damage to DNA as well as oxidative injury caused by reactive oxygen species. Oxidative injury has been shown to play an important role in retinal degeneration including age-related retinal disease [34–36], and it is possible that this is one of the mechanisms leading to the accelerated retinal degradation observed in the SIRT6 KO mice. Interestingly, other sirtuins, and especially Sirt1, have been shown to play a role in genetically determined and age-related retinal degeneration in rodent models [37–39] and activation of Sirt1 by oral supplementation of a SIRT1 activator, resveratrol, was shown to attenuate light-induced retinal injury and age-related retinal dysfunction in mice and rats [39, 40].

Taken together, these findings demonstrate again that SIRT6 may play a role in age-related metabolic and ocular changes, and raises the possibility that this protein may serve as a novel therapeutic target.

## Conclusions

The primary goal of this study was to examine the effects of SIRT6 deficiency in a mixed genetic background in order to abolish the potential strain specific dependent biased phenotypes and to allow long term follow-up into adulthood. Indeed, using this new model, we were able to show the effect of SIRT6 in adult mice and solve one of the main limitations of previous SIRT6 KO models, namely that of premature death at one month of age. The present study reveals that part of the severe metabolic phenotypes caused by SIRT6 deficiency are not strain specific, such as the higher glucose uptake in mice. However, some characteristics such as shortened lifespan are much less severe in this mixed background and, importantly, gender specific. The long-term evaluation of retinal function and structure afforded by this model suggests that absence of Sirt6 may accelerate retinal dysfunction and ageing.

## Supporting information

S1 Fig SIRT6 protein level. Western blot analysis for SIRT6 protein expression in WT and KO mice.
(PDF)

S2 Fig Loss of subcutaneous fat in SIRT6 deficient mice. Hematoxylin and eosin staining of skin sections from WT and KO male mice, showing an acute loss of subcutaneous fat in the absence of SIRT6.
(PDF)

S3 Fig GLUT1 protein levels. Flow cytometry analysis of GLUT1 protein levels in cell membrane of WT HET and KO cell MEFs.
(PDF)

S4 Fig Thinning of the Ganglion Cell Layer (GCL) in SIRT6 deficient mice with age. Thickness of the GCL was significantly lower in KO mice as compared with WT littermates throughout the experiment. However, measurement of linear density of ganglion cell nuclei did not show a difference between WT and KO mice: number of nuclei decreased in both experimental groups over time.
(PDF)
