# Supplementary figures and images for: Characterization of physiological defects in adult SIRT6-/- mice
Source: PLoS One. 2017 Apr 27;12(4):e0176371. doi: 10.1371/journal.pone.0176371 (PMC5407791; doi:10.1371/journal.pone.0176371)

S1 Fig.

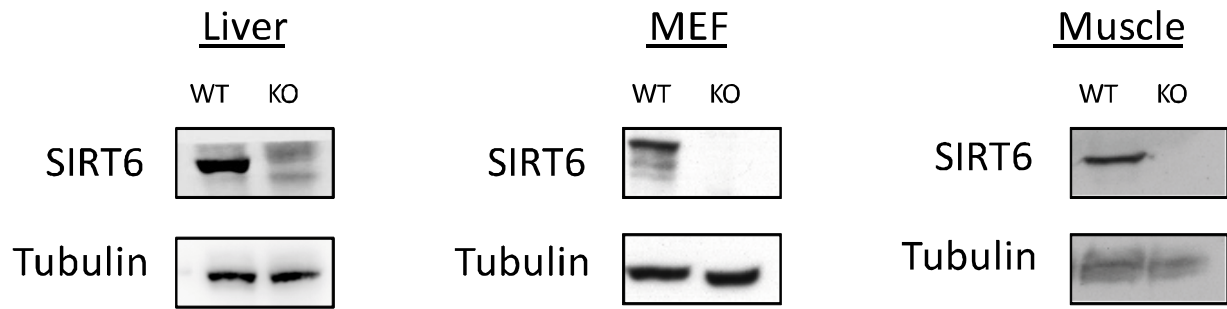

S1 Fig. Western blot analysis for SIRT6 expression in WT and KO mice.

Supplement: S1 Fig — Western blot analysis for SIRT6 protein expression in WT and KO mice. (PDF) [file pone.0176371.s001.pdf]
