# Supplementary material for: Characterization of physiological defects in adult SIRT6-/- mice
Source: PLoS One. 2017 Apr 27;12(4):e0176371. doi: 10.1371/journal.pone.0176371 (PMC5407791; doi:10.1371/journal.pone.0176371)
Supplement: S2 Fig — Hematoxylin and eosin staining of skin sections from WT and KO male mice, showing an acute loss of subcutaneous fat in the absence of SIRT6. (PDF) [file pone.0176371.s002.pdf]

S2 Fig.

WT KO

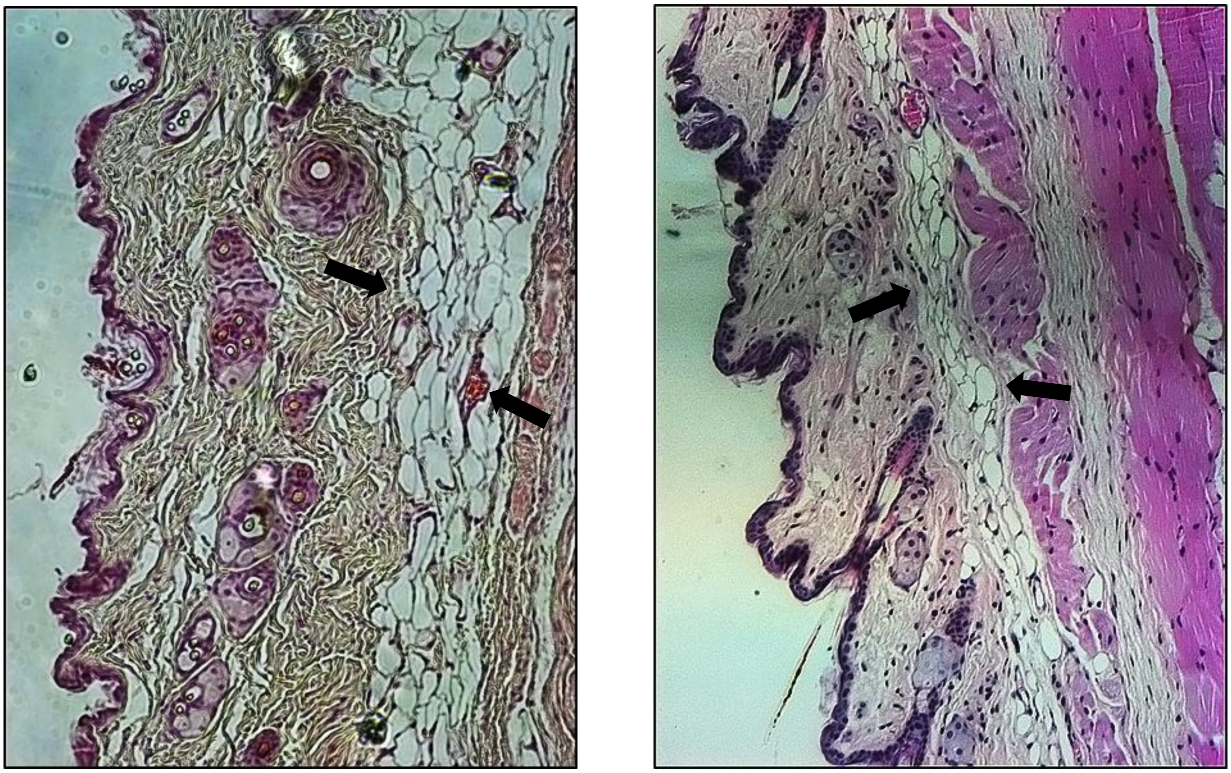

S2 Fig. Loss of subcutaneous fat in SIRT6 deficient mice. Hematoxylin and eosin staining of skin sections from WT and KO male mice, showing an acute loss of subcutaneous fat in the absence of SIRT6.
