# Supplementary material for: Characterization of physiological defects in adult SIRT6-/- mice
Source: PLoS One. 2017 Apr 27;12(4):e0176371. doi: 10.1371/journal.pone.0176371 (PMC5407791; doi:10.1371/journal.pone.0176371)
Supplement: S3 Fig — Flow cytometry analysis of GLUT1 protein levels in cell membrane of WT HET and KO cell MEFs. (PDF) [file pone.0176371.s003.pdf]

S3 Fig.

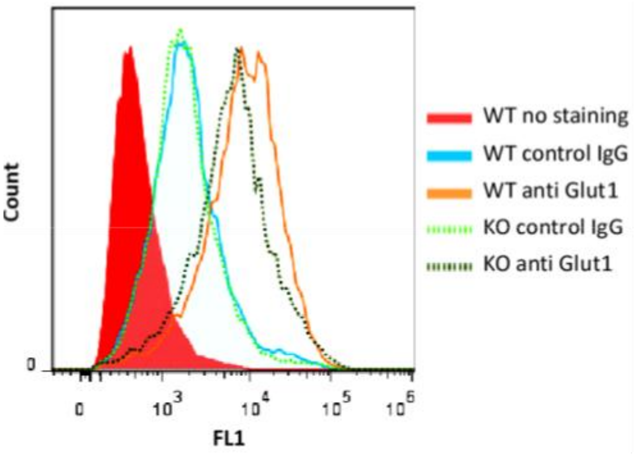

S3 Fig. Flow cytometry analysis of GLUT1 protein levels in cell membrane of WT HET and KO cell MEFs.
