# Supplementary material for: Characterization of physiological defects in adult SIRT6-/- mice
Source: PLoS One. 2017 Apr 27;12(4):e0176371. doi: 10.1371/journal.pone.0176371 (PMC5407791; doi:10.1371/journal.pone.0176371)
Supplement: S4 Fig — Thickness of the GCL was significantly lower in KO mice as compared with WT littermates throughout the experiment. However, measurement of linear density of ganglion cell nuclei did not show a difference between WT and KO mice: number of nuclei decreased in both experimental groups over time. (PDF) [file pone.0176371.s004.pdf]

S4 Fig.

.

A.

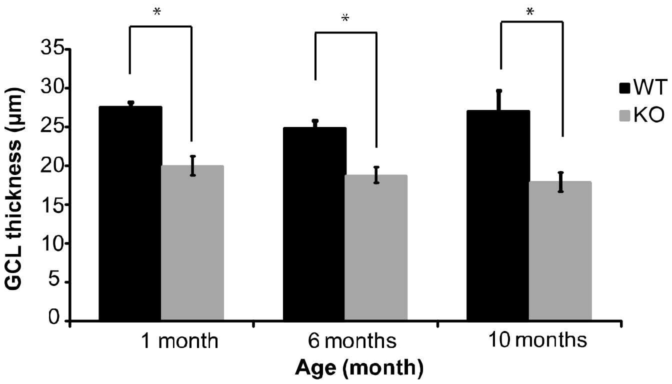

B.

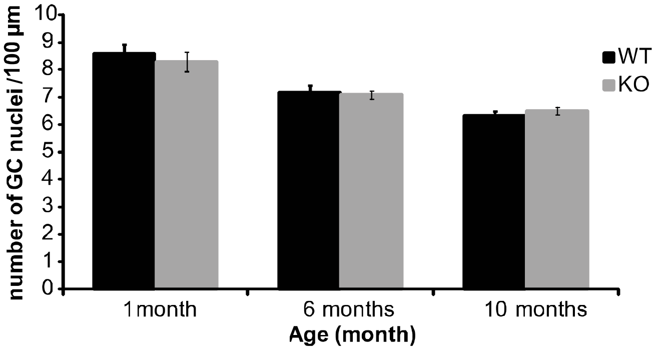

S4 Fig. Thinning of the Ganglion Cell Layer (GCL) in SIRT6 deficient mice with age. Thickness of the GCL was significantly lower in KO mice as compared with WT littermates throughout the experiment. However, measurement of linear density of ganglion cell nuclei did not show a difference between WT and KO mice: number of nuclei decreased in both experimental groups over time.
